# Use of Medicinal Plants and Natural Products for Treatment of Osteoporosis and Its Complications

**DOI:** 10.1155/2013/764701

**Published:** 2013-02-10

**Authors:** Ima Nirwana Soelaiman, Srijit Das, Ahmad Nazrun Shuid, Huanbiao Mo, Norazlina Mohamed

**Affiliations:** ^1^Department of Pharmacology, Faculty of Medicine, Universiti Kebangsaan Malaysia, 50300 Kuala Lumpur, Malaysia; ^2^Department of Anatomy, Faculty of Medicine, Universiti Kebangsaan Malaysia, 50300 Kuala Lumpur, Malaysia; ^3^Department of Nutrition & Food Sciences, Texas Woman's University, Denton, TX 76204, USA

Osteoporosis is characterized by low bone mass with susceptibility to fracture. It may only be discovered when fractures of the osteoporotic bone have occurred. Therefore, prevention and early treatment of osteoporosis are important to avoid its complications. Some of the commercially available antiosteoporotic drugs are associated with serious side effects. Research is ongoing to find alternative antiosteoporotic agents with comparable effectiveness but minimal side effects. It is hoped that the increased ethnobotanical and ethnopharmacological research may pave the way to the development of novel drug candidates. In this special issue, 28 papers were published addressing various aspects of natural products on osteoporosis and its complications. Of these, 7 were review articles and the rest were original articles. All the published papers contribute significantly towards providing scientific evidence for future use in research and clinical settings. 

Amongst the natural products, vitamin E, in particular tocotrienol, was the most studied, whereby 7 papers (4 original articles and 3 review articles) were published. The topics discussed were wide-ranging, including effects on bone structure and strength, mechanisms of action, and effects on osteoporotic fracture healing. S. Abdul-Majeed et al. reported on the effects of a combination of tocotrienol derived from annatto beans and lovastatin on bone resorption and formation indices. N. A. Manan et al. reported an *in vivo* study on the effects of *γ*-tocotrienol on osteoblasts. In conclusion, vitamin E of the tocotrienol type was shown to protect against experimental osteoporosis, whereby the mechanisms of these effects may be via its antioxidant or mevalonate suppressor properties, or both.

Other natural herbal products discussed were *Eurycoma longifola, Labisia pumila*, virgin coconut oil, *Cosmos caudatus,* curcumin, *Nigella sativa*, *Sambucus nigra,* and *Trifolium sp.* These were mainly extracts from the whole plant or specific parts of the plants, such as the fruit, leaves, or roots. However, S.-W. Choi et al. reported on fisetin, an alkaloid found in several different plants, where they described the mechanisms of the osteoclast inhibitory effects of fisetin. In general, these studies found encouraging effects of these natural products on bone metabolism, structure, and strength, as well as in improving the rate of osteoporotic fracture healing. However, further studies are needed to confirm these effects as well as to elucidate the precise mechanisms of action.

The effects of Chinese herbal products on osteoporosis were well represented in four papers. These studies included *in vivo* animal studies, *in vitro* cell culture studies, as well as a population-based study on usage of Chinese herbal medication in Taiwan. One study used the fermented product from Chinese herbal medication. In general, the animal studies showed that the Chinese medications studied had positive effects on bone. The population based-study identified patterns of Chinese herbal products used for the treatment of osteoporosis. However, further research is required to fully elucidate the efficacy and safety of these Chinese herbal products.

Overall, this special issue described studies on the effects of a plethora of natural products on different aspects of osteoporosis and osteoporotic fracture healing. These studies contribute greatly to the existing knowledge regarding prevention and treatment options for osteoporosis. It is anticipated that some of these products may be further developed into pharmacotherapy for osteoporosis and osteoporotic fracture healing.

*Ima Nirwana Soelaiman* *Ima Nirwana Soelaiman* *Srijit Das* *Srijit Das* *Ahmad Nazrun Shuid* *Ahmad Nazrun Shuid* *Huanbiao Mo* *Huanbiao Mo* *Norazlina Mohamed* *Norazlina Mohamed*

